# A green approach for the synthesis of α-Fe_2_O_3_ nanoparticles from *Gardenia resinifera* plant and it's *In vitro* hyperthermia application

**DOI:** 10.1016/j.heliyon.2019.e02044

**Published:** 2019-07-10

**Authors:** V.C. Karade, S.B. Parit, V.V. Dawkar, R.S. Devan, R.J. Choudhary, V.V. Kedge, N.V. Pawar, J.H. Kim, A.D. Chougale

**Affiliations:** a Optoelectronic Convergence Research Center, Department of Materials Science and Engineering, Chonnam National University, Gwangju, 500-757, South Korea; b Department of Chemistry, The New College, Shivaji University, Kolhapur, 416012, India; c Biochemical Science Division, CSIR-National Chemical Laboratory, CSIR, Pune, India; d Biotechnology and Pharma Division, MITCON Foundation, Shivajinagar, Pune 411005, India; e Discipline of Metallurgy Engineering and Materials Science, Indian Institute of Technology Indore, Simrol, Indore, 453552, India; f UGC-DAE Consortium for Scientific Research, Khandwa Road, Indore 452001, India; g Regrow, Regenerative Medical Services Pvt. Ltd, Lonavala, India; h Department of Botany, The New College, Shivaji University, Kolhapur, 416012, India

**Keywords:** Materials science, Nanotechnology, Green synthesis, Magnetic materials, Hyperthermia, Biomaterials

## Abstract

The *Gardenia*, traditional medicinal plant used from ancient time to increase appetite and other medicinal uses has been employed for the synthesis of superparamagnetic α-Fe_2_O_3_ nanoparticles (NPs). The plant extracts unveiled its bifunctional nature through the reducing ferric ions by phenolic groups and capping nature through the –OH bonding over the NPs surface. The prepared NPs exhibits α-Fe_2_O_3_ phase among iron oxides and spherical morphology with an average size around 5 nm. The magnetic measurements proved the superparamagnetic behavior of NPs with non-saturating M_S_ value of 8.5 emu/g at room temperature (300 K). Further, the hyperthermia study reveals, the NPs achieved a temperature of 40 °C and 43 °C within 6 min and reaches up to 43 °C and 45 °C within 10 min only for 5 μg/mL and 10 μg/mL concentrations respectively. Based on the heating profile of NPs, the SAR values (167.7 Oe, 300 MHz) calculated and are found to be around 62.75 W/g and 24.38 W/g for 5 μg/mL and 10 μg/mL NPs concentrations respectively. Subsequently, these have been used for toxicity assays, which presented enhanced cytotoxic effects on human mesenchymal cells lines proving them as a potential candidate for the biomedical applications.

## 1 Introduction

In recent years, magnetic and iron oxide-based nanoparticles (NPs) have been attracting great interest in diverse fields, due to their remarkable physio-chemical properties at the nanoscale [1, 2]. Specifically, the properties like biocompatibility of iron oxide NPs produced some abundant *In-vivo* applications such as contrast agent in magnetic resonance imaging (MRI), cells separation, and drug delivery field [3, 4, 5, 6]. In addition, the ability to generate heat in the presence of alternating (AC) magnetic field makes iron oxide NPs as a promising material for hyperthermia applications.

Among different phases of iron oxides, the widely used and most studied iron oxides are hematite (α-Fe_2_O_3_), maghemite (γ- Fe_2_O_3_) and magnetite (Fe_3_O_4_). Although some of them exhibit relatively poor magnetic properties, owing to its low cost and high resistivity offered towards the corrosion, maghemite can be considered as an alternative option for magnetite. The hematite is one of the most stable phase in natural environment and hence it extensively used in other non-bio applications such as catalysis, sensing etc. [7, 8]. Based on these applications different physical and chemicals techniques such as sono-chemical, co-precipitation, sol-gel and others have been developed and employed to synthesize the NPs [9]. These synthesis process sometimes include the use of hazardous chemicals and/or generation of hazardous by-products [10, 11]. Moreover, certain molecules or chemical agents are deliberately used for the capping of the NPs surface makes these protocols more expensive [12]. Accordingly, there is a need to develop an alternative option for these methods which can overcome the above difficulties.

Recently green synthesis methods have received increasing attention due to their eco-friendly and therapeutic applicability aspects [13, 14]. These green approaches have some additional advantages like simplistic process, economic feasibility and less waste generation. Subsequently, various naturally available resources such as plant products, magneto-tactic bacteria, algae, yeast and viruses are used for the green synthesis of NPs. Most of the *In vitro* or *In vivo* approaches gives spherical shaped NPs but with wide size distribution based on the reducing environment [10]. However, the plant-based extracts give the impression of being the best reducing or capping agents for their easy availability, appropriateness for bulk production and making non-toxic waste compared to other microbial entities extracts [15]. The additional advantage of medicinal plant extract mediated NPs synthesis is that, the NPs surface can be stabilized by the harmless plant components, thus these NPs shows the nontoxic behavior towards the normal human cells.

In the present study, the NPs are synthesized using medicinal plant *Gardenia resinifera* extract and characterized by different analytical techniques. Further, they have been used to study the cytotoxicity assay and hyperthermia activity in AC magnetic field.

## 2 Experimental

### 2.1 Preparation of *G. resinifera* plant extract

The fresh *G. resinifera* plant leaves were collected, washed, air-dried and milled to get the fine powder. The powder (10 gm) was soaked in methanol at room temperature (RT) for 2 days and centrifuged at 10000 rpm for 10 min. Finally, the extract was filtered through 0.45 μM filter membrane and stored at 4 °C until further use.

### 2.2 Synthesis of NPs

The NPs were synthesized by addition of *G. resinifera* extract to FeCl_3_.6H_2_O (0.4 mM) in a 3:1 volume ratio with addition of sodium acetate (2 M) at RT and heated at 80 °C for 2 h. The prepared α-Fe_2_O_3_ NPs were washed magnetically with water and air-dried.

### 2.3 Characterization of *G. resinifera* NPs by various techniques

The structural and morphological studies of green synthesized NPs were characterized by X-ray diffraction (XRD, Bruker AXS) measurements (EDS, Oxford X-Max 50, German) and Transmission electron microscopy (TEM, JEM-2100HR, Japan). The characteristic interaction among the biomolecule found in extract and NPs were evaluated by Fourier transform infrared spectroscopy (FT-IR, Shimadzu). All the magnetization measurements were performed for the powder samples. The RT magnetization curve at 300 K was measured with MPMS-7T SQUID-VSM QUANTUM-DESIGN vibrating sample magnetometer. The temperature dependent magnetic behavior i.e. Zero-field-cooled (ZFC) and field-cooled (FC) magnetization measurements were performed in the temperature range 2 K–350 K in an applied magnetic field of 100 Oe.

### 2.4 Cytotoxicity and flow cytometry assay of NPs

I The cells were grown in DMEM (Dulbecco's Modified Eagle Medium) supplemented with 10% v/v fetal bovine serum, kanamycin (0.1 mg/mL), penicillin G (100 U/mL) and sodium bicarbonate (1.5 mg/mL) at 37 °C in a 5% CO_2_ atmosphere. The cells were incubated with DMEM (1×10^4^ cells/mL) for 24 h in 96-well microtiter plate. After 24 h, the cells were given fresh media with different proportions of sterile NPs (0.2, 0.4, 0.6, 0.8 and 1.0 mg/mL). At the last the whole medium was incubated at 37 °C in a 5% CO_2_ atmosphere for 24 h (and 48 h).
II Fluorescence-Activated Cell Sorter (FACS) in a BD (Becton, Dickinson) FACS CANTO was employed for the cytotoxic study. In this study, fluorescence excitation was carried out by using an argon-ion and a red LASER of 488 and 632 nm, respectively. The fluorescence emission was recorded by using corresponding detectors. Approximately 1 million cells were stained with predefined antibody cocktails (CD 90^+^, CD 73^+^). The stained cells were incubated in the dark for 20 min at RT, washed with FACS flow buffer (BD Biosciences) and re-suspended in FACS flow buffer and analyzed on a BD FACS CANTO. Data acquisition and analysis were accomplished by using BD FACS Diva software (BD Biosciences).

### *2.5* *In vitro* hyperthermia study of NPs

*In vitro* hyperthermia study for green synthesized hematite NPs were carried out in an induction heating unit (Easy Heat 8310, Ambrell; UK) having diameter 6 cm and 4 turns of the heating coil. To maintain the constant surrounding temperature, the water was circulated in coils. The NPs were continuously sonicated for 20 min in bath-sonicator prior to the hyperthermia measurement. The NPs with 5 μg/mL and 10 μg/mL concentrations were placed at the center of the coil and subsequently 300 MHz frequency was applied with an amplitude of 167.7 Oe for the desired time.

For the conducted experiments, the magnetic field was calculated from following equation,

(1) $$H=\;\frac{1.25*n*i}{L}\;Oe$$

Where n-number of turns; i-applied current and L-diameter of the turn for the coil.

The calculated value of the magnetic field (H) at 100 Amp was 167.7 Oe (equivalent to 13.34 kA/m).

### 2.6 SAR value calculations

The specific absorption rate (SAR) is the amount of heat produced by NPs in the presence of applied AC magnetic field is calculated by the following equation,

(2) $$SAR=c\;\frac{\text{Δ}T}{\text{Δ}t}\frac{1}{m_{magn}}$$

Where c is the sample-specific heat capacity of NPs in water.

The heat-capacity of both the samples was negligible at low concentration of NPs and thus a heat capacity for water (4.18 J/g/K) was taken as the sample's heat capacity. The ΔT/Δt is the preliminary slope of temperature Vs time curve and value of m_mag_ was considered as the number of NPs per total amount of NPs and water [16].

## 3 Results and discussion

**Fig. 1:**
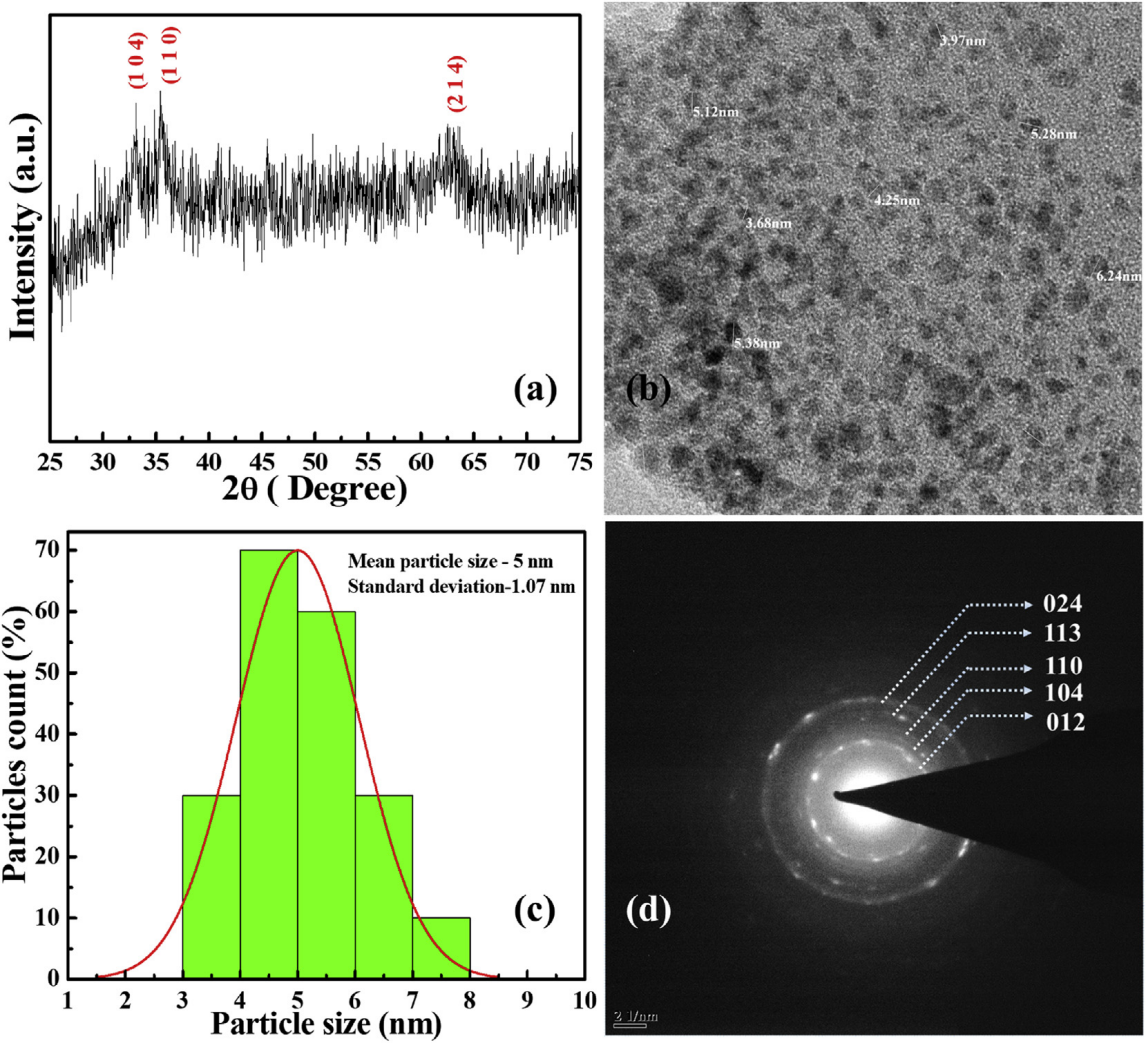
(a) X-ray diffraction pattern of α-Fe_2_O_3_ NPs, (b) high magnification TEM image of NPs, (c) particle size distribution of NPs determined from HR-TEM image and (d) SEAD pattern of α-Fe_2_O_3_ NPs.

The X-ray diffractogram of green synthesized NPs shows the pure α-Fe_2_O_3_ phase of iron oxide (Fig. 1a) matched with corresponding JCPDS No. 01-089-2810. The diffraction peaks clearly visible at 2θ values of 33.1, 35.53 and 62.53 in the X-ray diffractogram were allocated to the lattice planes of (104), (110) and (214), respectively. To evaluate the size and shape of green-synthesized NPs, high-resolution transmission electron microscope (HR-TEM) study was performed. The HR-TEM image in the magnified view shows the NPs have spherical morphology (Fig. 1b). The particle size histogram was obtained based on the HR-TEM image and the statistical analysis was performed. The histogram shows that the NPs exhibits nearly homogeneous size distribution with size extending among 3–8 nm. The mean size distribution discloses the average size of NPs is around 5 nm with a standard deviation of 1.07 nm (Fig. 1c).

The corresponding selected area electron diffraction (SEAD) pattern of NPs exhibited concentrated rings for the lattice planes of (104), (110) and others were consistent with XRD results (Fig. 1d).

**Fig. 2:**
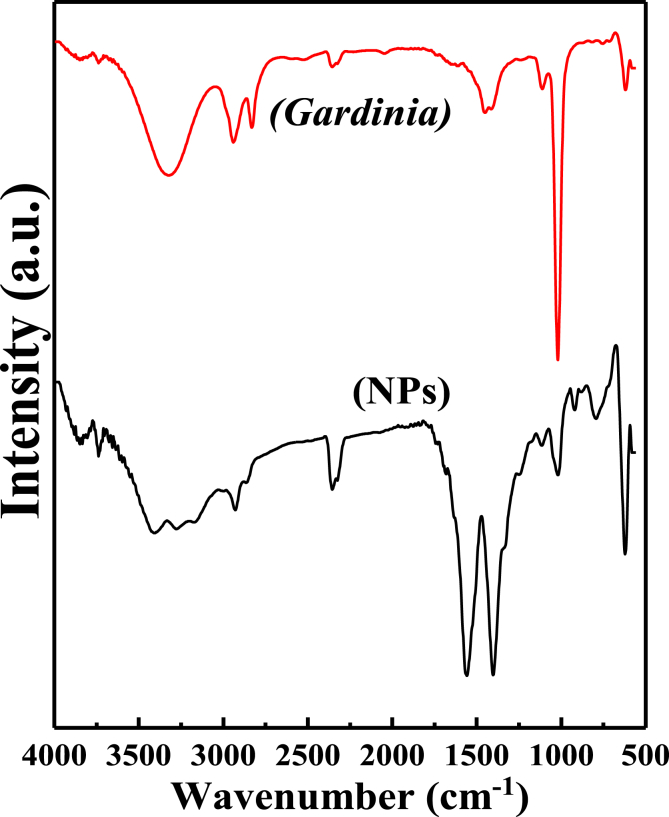
FT-IR spectra of NPs and *Gardenia resinifera* plant extract.

The Fourier-transform infrared spectroscopy (FT-IR) characterization of NPs and plant extract revealed the characteristic functional groups found in both of them (Fig. 2). The absorption band near 622 cm^−1^ assigned to the vibration of the Fe–O bond in iron oxide [17]. In the FT-IR spectra of NPs and extract, the wide-range band observed at 3300-3400 cm^−1^ of O–H stretching vibration may results from the phenolic hydroxyl group present in polyphenolic compounds [18, 19]. The bands at 1550-1600 cm^−1^, 1400 cm^−1^ and 1026.60 cm^−1^ are ascribed to the stretching vibration of C=O, bending vibration of C–H and stretching vibration of C–N bonds, respectively, confirming the presence of phenolic acids, terpenoids-phenols and aliphatic amines respectively [19]. The presence of identical bands found in plant extract and NPs, confirm that these bands attributed to NPs due to plant extract functional groups. Furthermore, this property validates that they may responsible for reducing ferric ions and capping the NPs surface.

**Fig. 3:**
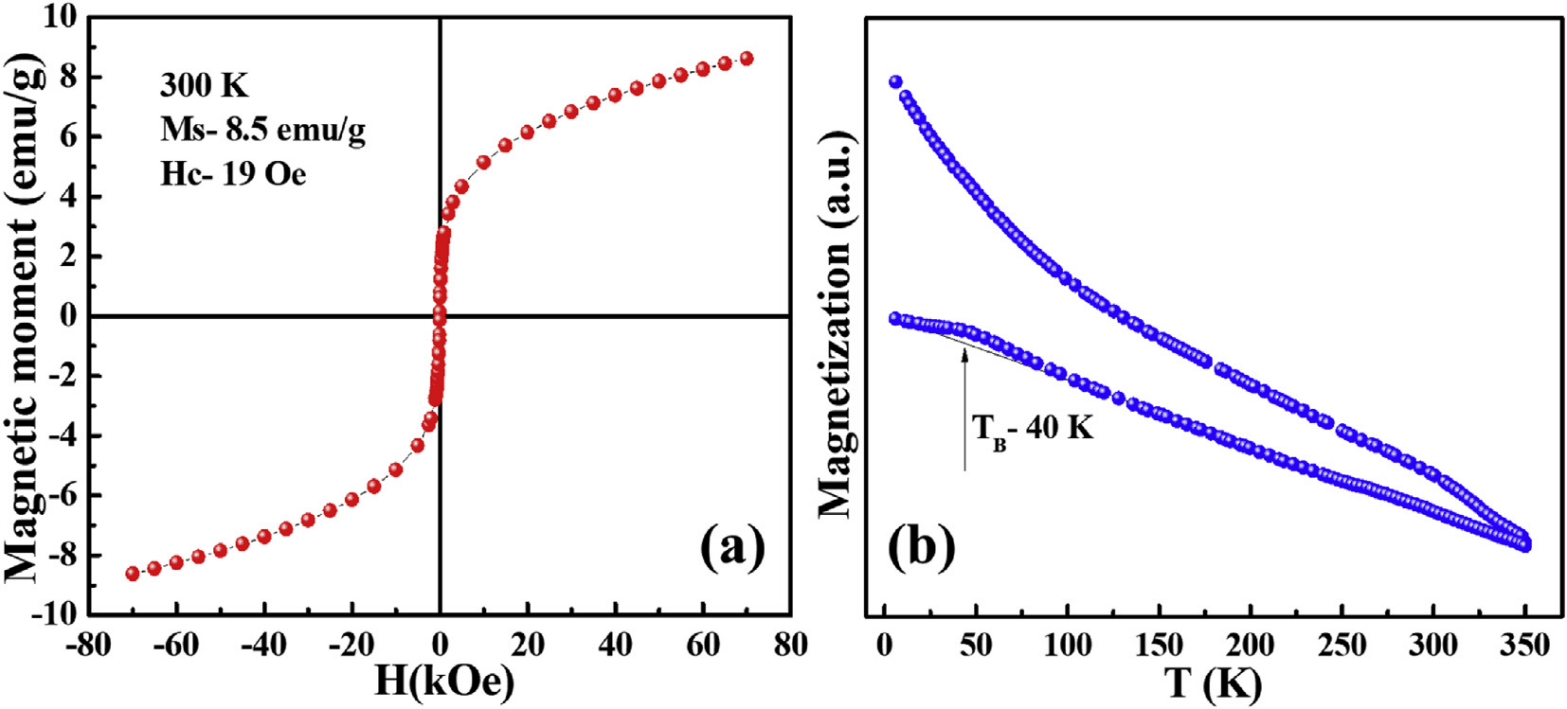
(a) RT (300 K) magnetization curve of α-Fe_2_O_3_ NPs traced with an applied magnetic field of 70 kOe and (b) temperature dependent magnetic behavior (ZFC-FC) of NPs measured at 100 Oe.

In the present M-H curve, the measured magnetic moment normalized to the total mass of the powder. The magnetization measurements performed at RT (300 K) of green synthesized NPs, exhibit nearly zero coercivity and the trace curve passes through the origin as shown in Fig. 3(a). This behavior specifies the superparamagnetic nature of NPs at RT with saturation magnetization (M_S_) value about 8.5 emu/g. The similar superparamagnetic behavior for hematite NPs was observed by Tadic et al. [20], they exhibit size around 8 nm for which M_S_ value was about 3.98 emu/g. The M_S_ value for bulk hematite is nearby 0.3 emu/g as reported by Teja et al. [21]. However, in the present work, the observed M_S_ value is higher than bulk ones as well as reported by Tadic et al. [20]. This high M_S_ value possibly comes from the exterior surface disordered spin, which may get easily orientated along the direction of the external applied magnetic field compared to core ones [20]. The non-saturating magnetization of NPs was also observed at a high applied field of 70 kOe, this non-saturating magnetization in hematite mainly observed due to the antiferromagnetic core and surface disordered spin from hematite. The similar behavior of non-saturating magnetization for high applied was noticed by Xia et al. [22] and Balaraju et al. [23]. The temperature dependent magnetization curves i.e. ZFC-FC curves were measured at 100 Oe and the results are depicted in Fig. 3(b). The maxima in the ZFC curve corresponds to the blocking temperature (T_B_) is found to be around 40 K. Below the T_B_ the ZFC curve decline abruptly, however up to 2 K the FC curve augmented progressively with decreasing temperature. This behavior in FC curve mainly attributed to the ferromagnetic interaction, the similar trend in FC curve was observed by Chakrabarty et al. [24].

**Fig. 4:**
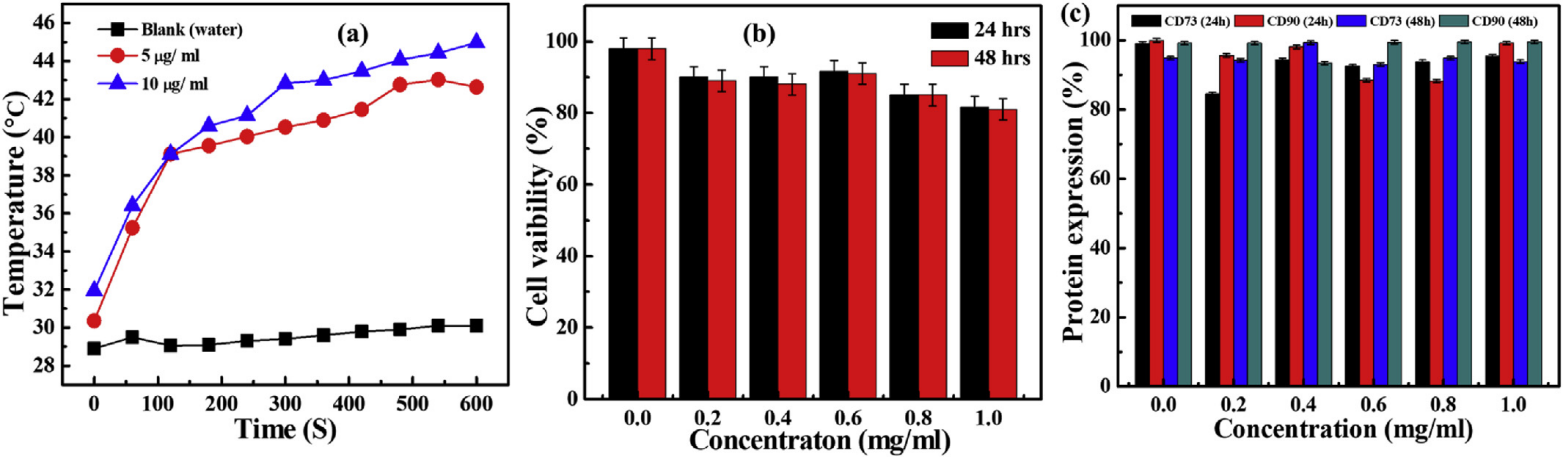
(a) Heating profile of α-Fe_2_O_3_ NPs measured as a function of time for 10 μg/mL and 5 μg/mL NPs concentrations and blank (water) respectively, (b) and (c) cell viability and flow cytometry assay of NPs at different concentrations for 24 hrs and 48 hrs respectively.

The external AC magnetic field of suitable frequency produces heat dissipation through the oscillation of the internal magnetic moment of NPs [25]. The temperature kinetic curves produced by the NPs represent the time-dependent temperature rise for both the samples (5 and 10 μg/mL) at an applied AC magnetic field of 300 MHz. The blank measurement of the suspension medium (water) was also measured and it shows negligible response with respect to time in the AC magnetic field. Thus, it's clear that, there was no role of apparatus intended for temperature rise in existing hyperthymia curves. In a recent study by Quinto et al. [26] presented that chemically synthesized Fe NPs modified with a hydrophobic group requires 20 min to achieve a temperature of 43 °C at 100 μg/mL concentration. However, in the present work the NPs synthesized by green route, achieved the temperature of 43 °C within 6 min and reaches up to 45 °C within 10 min only at 10 μg/mL concentration (Fig. 4a). Besides, even at low concentration (5 μg/mL), the temperature was augmented with respect to time and reached at 40 °C within 6 min and subsequently reached up to 43 °C within 9 min. Based on the heating profile of NPs, the SAR values were found to be ∼24.38 W/g and ∼62.75 W/g for 10 μg/mL and 5 μg/mL NPs concentrations, respectively. The polyphenolic compounds from *G. resinifera* plant extract, which may responsible for capping behavior of relatively small sized NPs mainly contributed to the significant enhancement in SAR values of green NPs. As the capping of NPs surface prevents agglomeration and it was reported that, the well dispersed superparamagnetic NPs boosts the hyperthermia effect through improved Brownian and Neel's spin relaxations [27].

The plant components are non-toxic, consequently their cumulative effect with NPs was significantly observed on human mesenchymal cells lines in the present work. The cell viability and surface expression assess were studied through flow cytometry done at different incubation time (24 and 48 h) and NPs concentrations. The obtained results from both studies are shown in Fig. 4b. The cytotoxic study displayed more than 82% and 81% of cells viability after incubation of NPs with human mesenchymal cells for 24 h and 48 h, respectively. They show noteworthy cell viability relative to control, even at high concentration of NPs (1 mg/mL). Similarly, the cell surface expressions for CD90 and CD73 markers were also found to be more than 90% at all concentrations (Fig. 4c), validating biocompatibility of NPs on the cells even at high expression levels.

## 4 Conclusions

In the present report, low cost and eco-friendly approach have been employed to synthesize the α-Fe_2_O_3_ iron oxide NPs concerning the biomedical applications. The *G. resinifera* plant extract based facile approach produce the nearly spherical NPs with size about 5 nm stabilized by polyphenols. Even though the NPs are small, they revealed superparamagnetic nature with non-saturating M_S_ value of 8.5 emu/g at RT. The heating profile of NPs measured with respect to time for different concentrations proved their heating ability in AC magnetic field. The NPs attained the temperature of 43 °C within just 10 min even at low concentration of 5 μg/mL with SAR value around 62.75 W/g for an applied AC field of 300 MHz and magnitude 167.7 Oe. Further synthesized NPs have been used for toxicity assays, which presented significant cytotoxic effects on human mesenchymal cells with more than 80% viability up to 48 h. The inclusive outcomes of the present work can deliver a promising footstep in low cost and eco-friendly approaches of bioinspired materials. As, the green synthesized NPs are not only biologically safe but also the potential candidate for forthcoming biomedical applications.

## Declarations

### Author contribution statement

A.D. Chougale: Conceived and designed the experiments; Analyzed and interpreted the data.

S.B. Parit: Performed the experiments; Wrote the paper.

R. J. Choudhary & V. V. Kedge: Performed the experiments.

V.C. Karade: Analyzed and interpreted the data; Wrote the paper.

V. V. Dawkar, N.V. Pawar, R. S. Devan & J. H. Kim: Contributed reagents, materials, analysis tools or data.

### Funding statement

This work was supported by the Human Resources Development program (No. 20194030202470) of the Korea Institute of Energy Technology Evaluation and Planning (KETEP) Grant funded by the Korean Government Ministry of Trade, Industry also by Priority Research Centers Program through the National Research Foundation of Korea (NRF) funded by the Ministry of Education, Science and Technology (2018R1A6A1A03024334).

### Competing interest statement

The authors declare no conflict of interest.

### Additional information

No additional information is available for this paper.
